# Towards more accurate measurement of edge to os distance in low-lying placenta using three dimensional transvaginal ultrasound: an innovative technique

**DOI:** 10.1186/s12884-018-2107-4

**Published:** 2018-12-04

**Authors:** Somayya M. Sadek, Reda A. Ahmad, Hytham Atia, Adel G. Abdullah

**Affiliations:** Obstetrics and Gynecology Department, Faculty of Medicine, Zagazig University, Zagazig, Egypt

**Keywords:** Low-lying, Placenta, Distance, Three-dimensional, Ultrasound

## Abstract

**Background:**

Measurement of edge to os distance (EOD) is essential to differentiate low-lying from normal placenta, and to plan for delivery. Till now, measurement by 2D TVS is the gold standard, however, its accuracy is questioned. In this study, we introduced an innovative technique for measurement of EOD using 3D TVS. Our aim was to compare EOD measurements of the standard 2D technique, to those of our innovative 3D technique, and to correlate the difference, if any, with placental site and internal os width.

**Methods:**

This study was conducted in the ultrasound unit of obstetrics and gynecology department, Zagazig University Hospitals, during the period from June 2014 to August 2017. Seventy six cases in whom the lower placental edge didn’t reach the internal os (IO), and the EOD was less than 35 mm, were included in the study. Placental location was identified by 2D transabdominal sonography then 2D TVS was used to measure the EOD in all cases. Our new technique was then applied to measure EOD by 3D TVS following stepwise manipulations of the orthogonal planes in multiplanar view. Width of IO was measured also in all cases.

**Results:**

The mean EOD measured by 3D TVS was significantly shorter than that measured using the 2D TVS. Anterolateral/posterolateral and lateral placentas were associated with high discrepancy in measurements between both methods, being the highest with lateral group. There was significant positive correlation between the IO width and the degree of difference between the EOD measured by both methods.

**Conclusions:**

Two dimensional TVS may not be accurate in EOD measurements in many cases of low-lying placentas, and 3D TVS may increase the accuracy of measurements in these cases. This new method is simple, precise and easily applied.

**Electronic supplementary material:**

The online version of this article (10.1186/s12884-018-2107-4) contains supplementary material, which is available to authorized users.

## Background

Placenta previa is a risky obstetric condition that often herald deleterious maternal and fetal outcomes. It is a relatively common problem complicating one of every 200 deliveries. This rate is prone for more increment with the rising cesarean delivery rate [1–3].

Most authors consider the diagnosis of previa when the lower placental edge is covering or reaching the internal os (IO), and it is defined as low-lying if the edge to os distance (EOD) is 1–20 mm [2, 4, 5]. However, some still consider these cases as previa [6], while others suggest EOD of 35 or even 40 mm to define the low-lying placenta [7]. Placenta previa has been commonly classified into major (overlapping or reaching the IO) and minor (within 2 cm from IO) types, or into four groups according to the EOD measured by TVS; grade I (more than 2 cm from os), grade II (11–20 mm), grade III (0–10 mm) and grade IV (Overlapping the os by any distance) [8].

This unfinished debate regarding the definition of previa and low-lying placenta is basically raised in concern to the anticipated progressively increased risk of antepartum bleeding as the placenta becomes closer to the IO, beside the need to define the relatively safe distance to allow vaginal delivery. It is agreed that cesarean delivery would be the ideal mode of delivery when the placenta is covering or within 10 mm from the IO. The majority still prefer cesarean delivery also when EOD is 11–20 mm [9], while some hypothesized the safety of vaginal delivery in such cases [8, 10, 11]. When the EOD is 20–35 mm, cases would deserve the attempt for vaginal delivery with caution after detailed counselling. Despite being safer than the previous 2 groups, they are still at increased risk of antepartum or postpartum hemorrhage [4, 12, 13].

When few millimeters may be critical in the diagnosis and management, there is a real need for a precise method to measure the EOD accurately. Two dimensional transvaginal sonography (2D TVS) is the routinely used and gold standard diagnostic method for evaluation of such cases with confirmed safety [14, 15], but its accuracy for such purpose was questioned by *Simon* et al. when significantly different measurements for the same EOD were reported by two sonographers. The three dimensional transvaginal sonography (3D TVS) evaluation was suggested as a more precise and objective method for EOD measurement [16].

In this study, we introduced an innovative technique for measurement of EOD using 3D TVS in cases with low-lying placenta. Our aim was to compare EOD measurements of the standard 2D technique, to those of our 3D technique, and to correlate the difference, if any, with placental site and IO width.

## Methods

This prospective observational study was conducted in the ultrasound unit of obstetrics and gynecology department, Zagazig University Hospitals, during the period from June 2014 to August 2017. Cases were recruited from those referred to our unit for transvaginal ultrasound scan to confirm or exclude suspected placenta previa during antenatal care. After approval by the local ethical committee of Zagazig University Hospitals (ZU-IRB#4961-3-6-2014) and oral consent, ultrasound examination was carried out for all cases using C1-5D curved abdominal probe and RIC5–9D three-dimensional endovaginal probe (Voluson E6, GE Medical Systems, Zipf, Austria).

Transabdominal sonography (TAS) was performed for these cases to localize the placenta in relation to uterine walls. Placental location was classified as direct anterior/posterior, anterolateral/ posterolateral and lateral. Then, 2D TVS examination followed, to determine the relation between the lower placental edge and the IO. The probe was introduced in the vagina gently and under sonographic live visualization till reaching the cervix without compressing it. Depth was adjusted to get the cervix together with a part of the lower uterine segment in which the lower placental edge was well visualized. A mid-sagittal view of the cervix was obtained by panning and rotational movements of the probe till the cervical canal was visualized from the IO (the upper point of the cervical canal) to the external os. In cases with placental edge not reaching the IO, the probe was rotated 90°to both sides, keeping the IO in view, then the shortest distance between the placental edge and the IO (EOD) was measured in millimeters using two points (straight line) [7]. All 2D sonographic examinations were performed by one expert sonographer (R.A.). All cases in whom the lower placental edge didn’t reach the IO, and the EOD was less than 35 mm, were included in the study [7].

We used G*Power software (version 3.1.9.2, Heinrich-Heine-Universitat, Dusseldorf, Germany) to calculate the sample size. Given there is no previous studies suggesting mean difference in EOD measurements between 2D TVS and 3D TVS, we calculated the required sample size sufficient for effect size d 0.4, α error 0.05 and power 95%. Least required sample size was 70 cases.

**Fig. 1:**
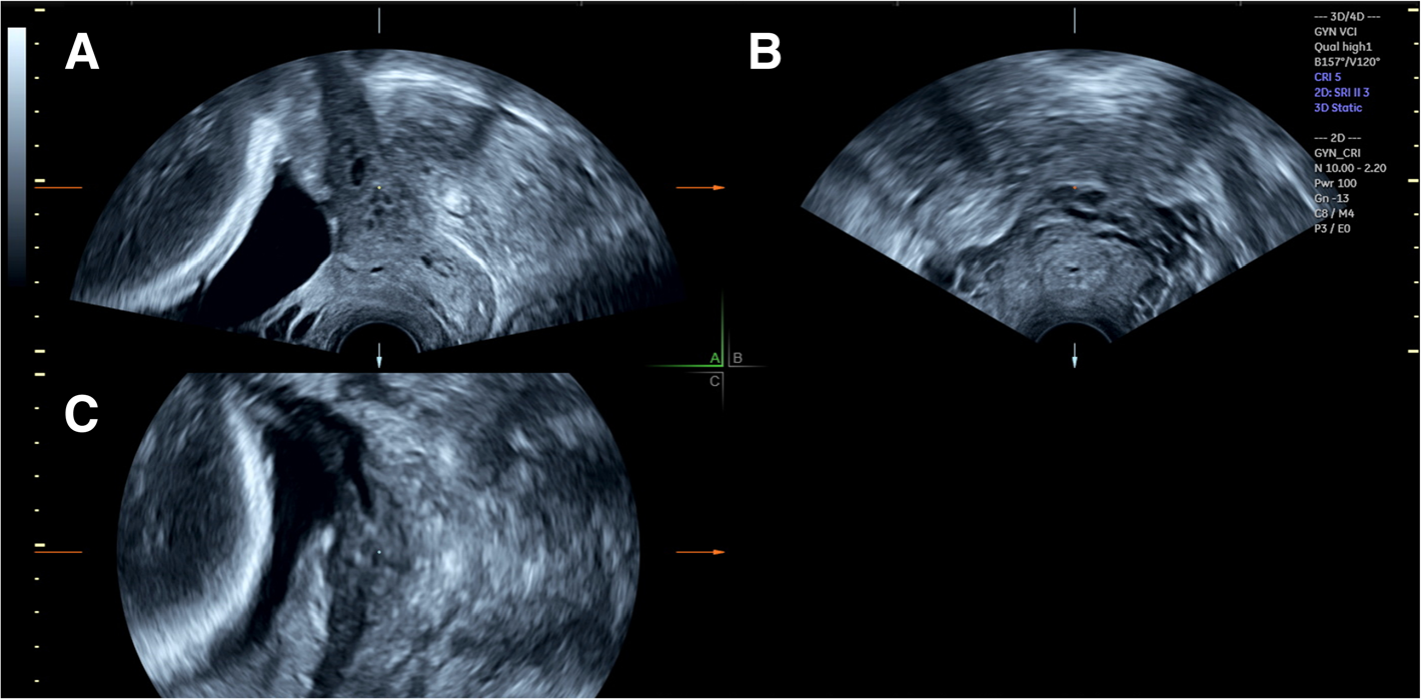
The multiplanar view of the initial dataset. The reference plane is the mid-sagittal view of the cervix (A)

**Fig. 2:**
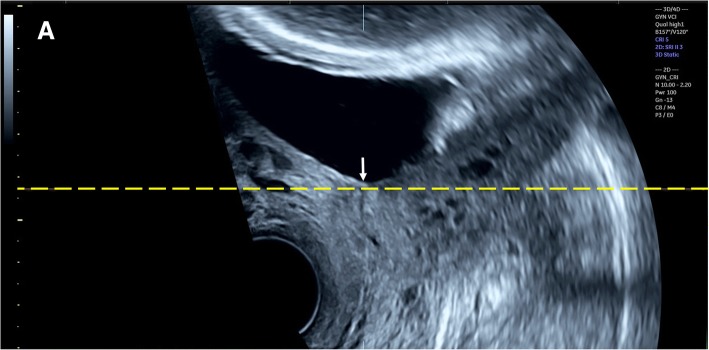
Plane A (sagittal view of the cervix) after manipulations (The IO was centered in the plane, the reference point was positioned at the IO then rotation around the z-axis to make the IO (arrow) at the lowest level in the lower uterine segment)

**Fig. 3:**
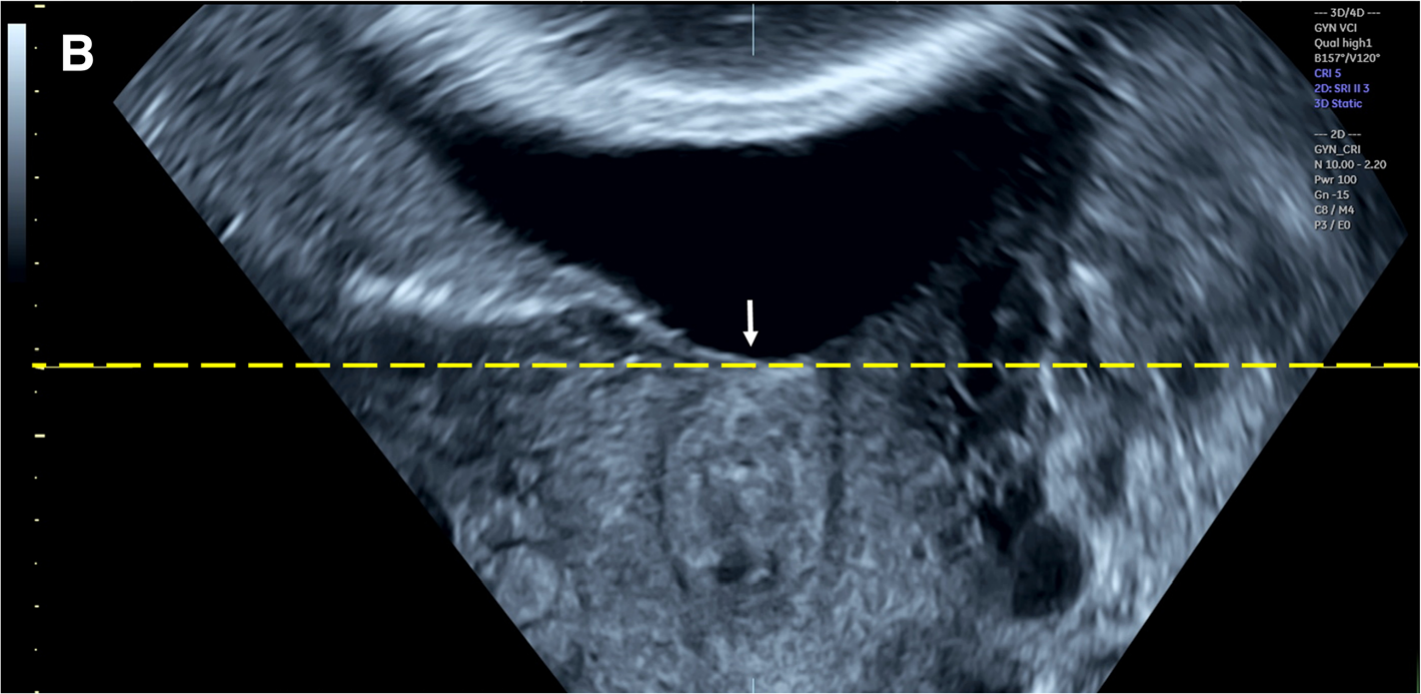
Plane B (coronal view of the cervix) after manipulations (The reference point was positioned in the middle of the upper edge of the cervical canal, then rotation around the z-axis to make the IO (arrow) at the lowest level in the lower segment)

**Fig. 4:**
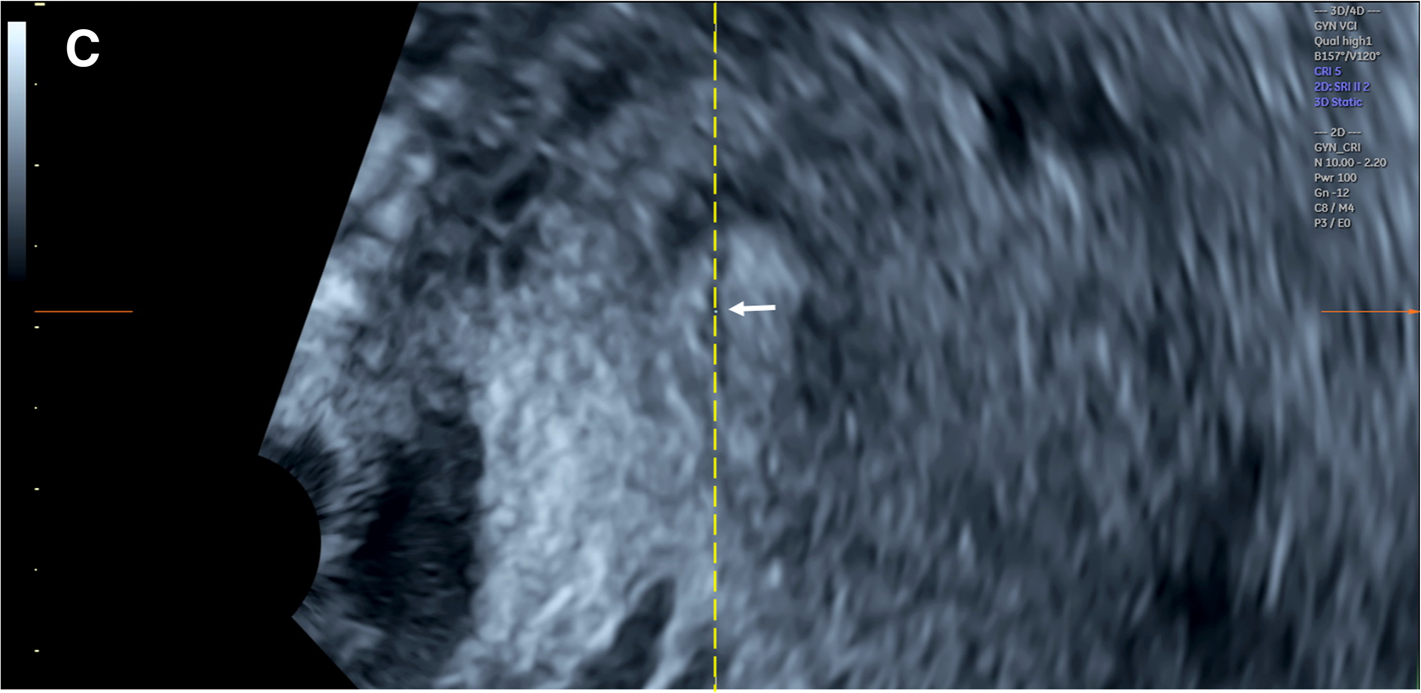
Plane C (axial view of the cervix) after manipulations (The reference point was positioned in the middle of the IO (arrow), then rotation around the z-axis to get the IO parallel to the y-axis)

**Fig. 5:**
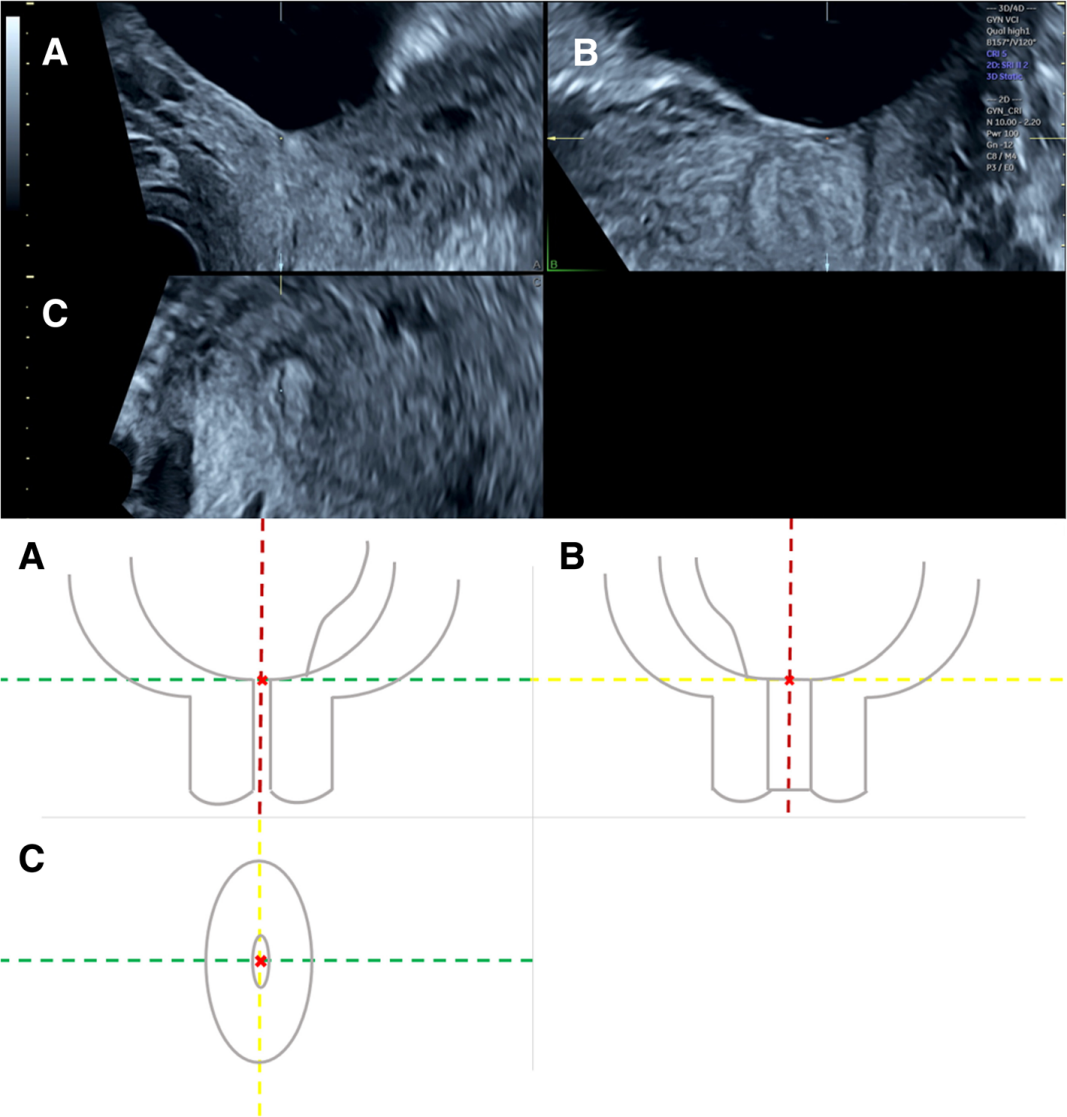
The multiplanar view after manipulations of the three planes, with an illustrative diagram: In reference to Plane A, the red line is the y-axis, the green line is the x-axis and the yellow line is the z-axis

**Fig. 6:**
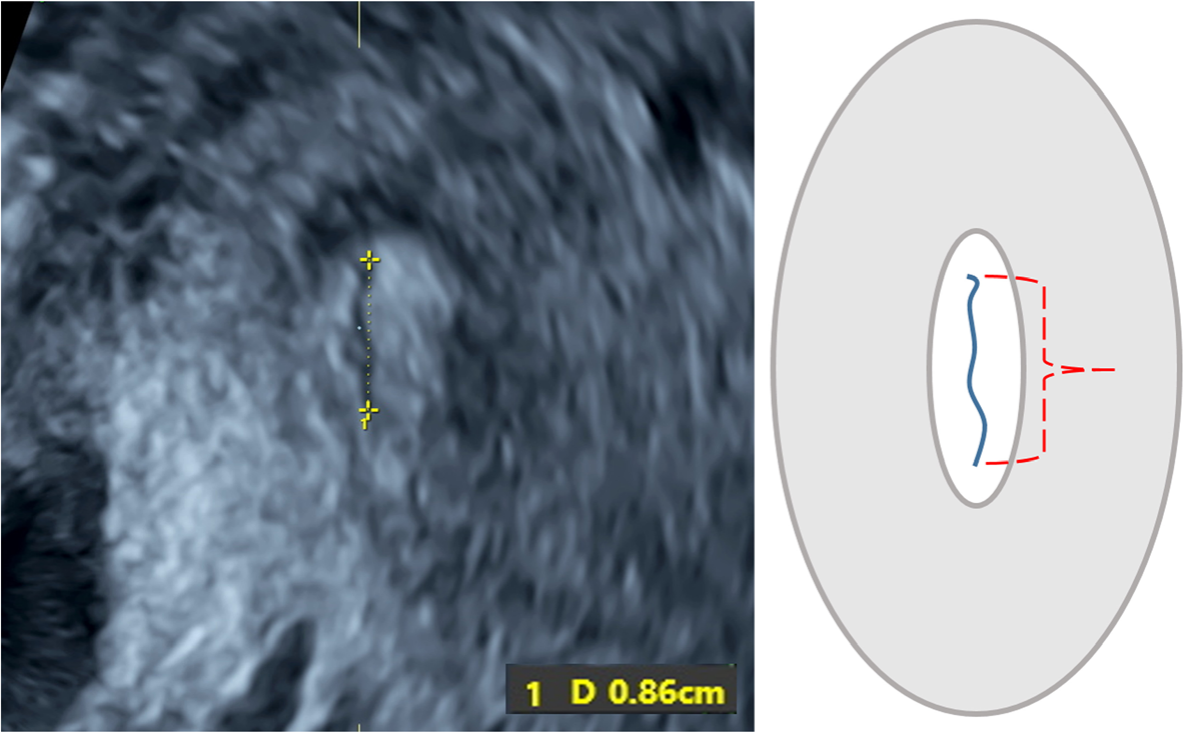
Measurement of the IO width in Plane C, with an illustrative diagram. The cervical mucosa appears as an oval hyperechogenic area with a slit inside representing the IO

**Fig. 7:**
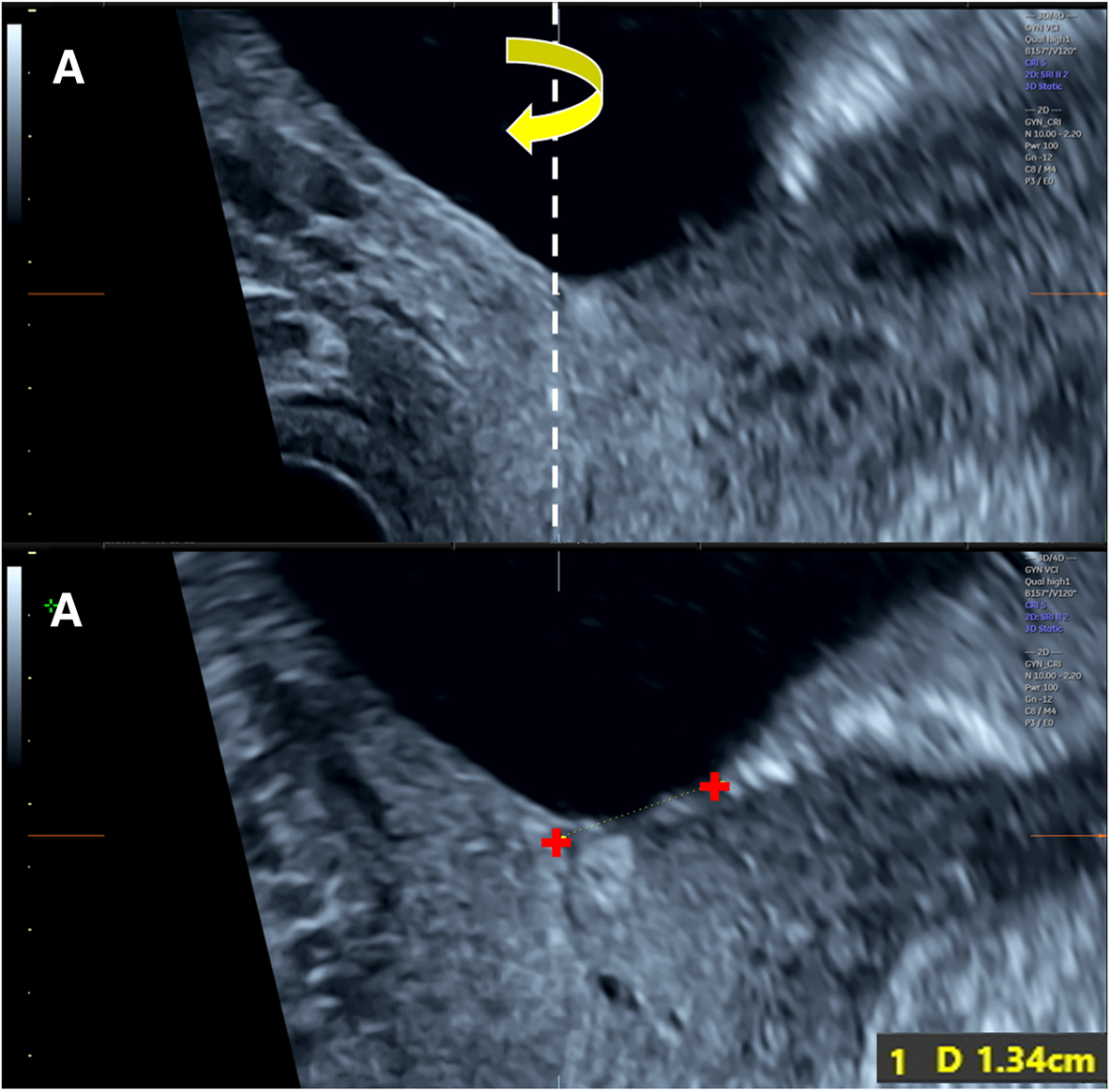
Plane A: Rotation 360° around the y-axis (arrow), then the shortest distance between the lower placental edge and the reference point (center of IO) was measured

Three-dimensional transvaginal sonography (3D TVS) was then performed for all cases included in the study by another expert sonographer (S.M.), who was blinded to the 2D TVS measurements. Before volume acquisition, a mid-sagittal view of the cervix was obtained, avoiding compression of the cervix or the lower segment. Examination was done in absence of uterine contractions and maternal and fetal movements. Volume was acquired with volume box and sweep angle adjusted to include at least the upper half of the cervix and the whole part of the lower uterine segment containing the lower placental edge (Quality: high 1). The multiplanar view of the initial dataset (Fig. 1) was manipulated in each case according to the following steps: (1) The IO was centered in Plane A, with magnification as needed. (2) The reference point was positioned at the IO in Plane A (upper point of the cervical canal). (3) Plane A was rotated around the z-axis to bring the reference point (i.e. IO) to the lowest level in the lower uterine segment (Fig. 2) (4) Plane B then represented the coronal view of the lower uterine segment and the upper part of the cervical canal, which appears as a rectangular hyperechogenic area. The reference point was repositioned in the middle of the upper edge of the cervical canal and the plane was rotated around the z-axis to bring the reference point to the lowest level in the lower segment (Fig. 3) (5) Plane C then represented the axial view of the cervix at the level of the IO, and for more confirmation, when the reference point was moved slightly above this level, the IO disappeared. In this plane, the cervical mucosa appeared nearly as an oval hyperechogenic area with a slit inside representing the IO. This slit was between the opposing anterior and posterior cervical walls at the upper end of the cervical canal (rectangular potential space). The reference point was repositioned in the middle of the IO. The plane was rotated around the z-axis to get the slit shaped IO parallel to the y-axis (Figs. 4 and 5) (6) Width of the internal os was measured in millimeters in Plane C (Fig. 6) (7) Plane A was rotated 360° around the y-axis. During rotation the lower placental edge became nearer to the reference point then moved away again. The shortest distance between the lower placental edge and the reference point (center of IO) was measured (using two points) in millimeters as the EOD (Fig. 7). Measurement of EOD by 3D TVS is summarized in Table 1.

**Table 1:** Summary of EOD measurement by 3D TVS

Volume acquisition	
1. Reference plane: The mid-sagittal view of the cervix.	
2. Acquisition box and angle: adjusted to include the upper part of the cervix and the lower uterine segment containing the lower placental edge.	
Volume display	
1. Plane A:	
a. The IO is centered in the plane.	
b. The reference point is positioned at the IO.	
c. Rotation around the z-axis to make the IO at the lowest level in the lower uterine segment.	
2. Plane B:	
a. The reference point is positioned in the middle of the upper edge of the cervical canal.	
b. Rotation around the z-axis to make the IO at the lowest level in the lower segment.	
3. Plane C:	
a. The reference point is positioned in the middle of the IO.	
b. Rotation around the z-axis to get the IO parallel to the y-axis.	
4. Plane A:	
a. Rotation 360° around the y-axis.	
b. Measure the shortest distance between the lower placental edge and the reference point (center of IO).	

Statistical analysis was performed using the following software products: SPSS© version 21 [IBM© Corp., Armonk, NY]. Shapiro–Wilk test was used to examine the numerical data for normality of distribution. Skewed data were presented as median and interquartile range (IQR). Normally distributed data were presented as mean ± standard deviation (SD). Categorical data were presented as number and percentage (%). Paired sample t-test was done to compare EOD measurements between 2D TVS and 3D TVS. Chi-Square test was used to compare patients grouped according to the EOD measured by both techniques. ANOVA was used to study the effect of placental location on the discrepancy in EOD measurements between both methods, Games- Howel Post Hoc test was used to test the degree of affection for every placental location. The relation between the same EOD discrepancy and internal os diameter measured by 3D TVS was tested by Pearson correlation coefficient.

## Results

**Fig. 8:**
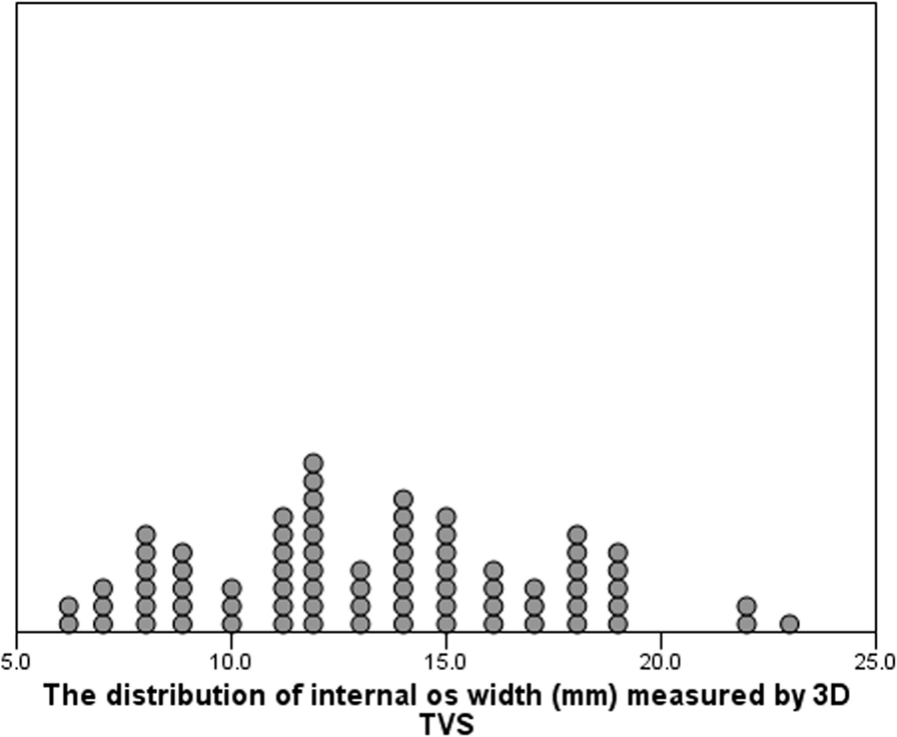
The distribution of internal os width (mm) measured by 3D TVS

During the study period, 76 cases were eligible for the study. Demographic data of the study group are shown in Table 2. Placental location was direct anterior/posterior in 6 cases (7.9%), anterolateral/ posterolateral in 61 cases (80.3%) and lateral in 9 cases (11.8%) (Table 3) (see also Additional file 1). The mean internal os width ranged from 6 to 23 mm with mean ± SD of 13.9 ± 5.5 mm (Fig. 8).

**Table 2:** Demographic data of the study group

Variable		Mean ± SD
Age (years)		29.7 ± 6.3
BMI (kg/m^2)^		26.1 ± 2.1
Gestational age at exam.(weeks)		32.7 ± 2.8
Variable		*n* (%)
Gestational age at exam.(weeks)	28- < 32	28 (36.8%)
	32- < 34	16 (21.1%)
	34- < 36	17 (22.4%)
	≥36	15 (19.7%)
Parity	0	6 (8%)
	1–2	35 (46%)
	≥3	35 (46%)
Previous cesarean	0	15 (19.7%)
	1–2	43 (56.7%)
	≥3	18 (23.6%)

**Table 3:** Placental location distribution

Placental location	*n* (%)
Anterior	3 (3.9%)
Anterior to the right	27 (35.5%)
Anterior to the left	14 (18.4%)
Posterior	3 (3.9%)
Posterior to the right	10 (13.2%)
Posterior to the left	10 (13.2%)
Right	7 (9.2%)
Left	2 (2.6%)

Despite EOD measured by 3D TVS was slightly longer in 7 cases (mean difference = − 1.45 mm, SD = − 1.21), paired sample *t* test revealed that the mean EOD measured by 3D TVS (*M* = 18.3, SD = 6.30) was significantly shorter than that measured using the 2D TVS (*M* = 24.26, SD = 7.08). We can be 95% confident that the true difference between these means is CI = [4.62, 7.29]. Cohen’s d was estimated 0.89, effect size = 0.4. This lead to dramatic increase in number of cases with EOD ≤ 10 mm [1 (1.3%) by 2D TVS vs 8 (10.5%) by 3D TVS], and cases with EOD 11–20 mm [22 (28.9%) by 2D TVS vs 42 (55.3%) by 3D TVS], *P* = 0.000 (Table 4).

**Table 4:** Comparison of the mean EOD as measured by 2D and 3D TVS

		2D TVS	3D TVS	Paired sample t-test
Edge to os distance	Mean distance	24.26 ± 7.08	18.3 ± 6.30	0.000
	Groups	*n* (%)	*n* (%)	Chi-Square test
	≤ 10 mm	1 (1.3%)	8 (10.5%)	0.000
	11–20 mm	22 (28.9%)	42 (55.3%)	
	21–30 mm	34 (44.7%)	23 (30.3%)	
	31–35 mm	19 (25%)	3 (3.9%)	

Patients were stratified according to the EOD by both methods

The ANOVA revealed a main effect of placental location on the degree of difference in measurements of EOD between 3D and 2D TVS, F (7, 68) = 4.122, *P* = 0.001 (Table 5). Anterolateral/posterolateral and lateral placentas were associated with high discrepancy in measurements between both methods, being the highest with lateral group (Table 6).

**Table 5:** Relation between the location of the placenta and the mean difference between 2D and 3D TVS EOD measurements

Placental location	Mean difference (2D-3D estimate)	One way ANOVA	Games- Howel Post Hoc test
Anterior	1 mm	0.001	1
Anterior to the right	5.3 mm		0.028
Anterior to the left	4.7 mm		0.250
Posterior	0.7 mm		1
Posterior to the right	7.3 mm		0.073
Posterior to the left	4.3 mm		0.137
Right	13.4 mm		0.005
Left	14.4 mm		0.387

**Table 6:** Relation between groups of placental location and the mean difference between 2D and 3D TVS EOD measurements

Placental location groups	Mean difference (2D-3D estimate)(mean ± SD) mm	One way ANOVA	Games- Howel Post Hoc test
Direct Anterior/posterior	0.85 ± 0.82	0.000	1
Anterolateral/posterolateral	5.33 ± 5.29		0.001
Lateral	13.59 ± 4.56		0.000

Pearson correlation coefficient revealed a significant positive correlation between the IO width and the degree of difference between the EOD measured by both methods, r (74) = 0.345, *P* = 0.001 (Table 7).

**Table 7:** Relation between the IO width and the difference in EOD measured by both 2D and 3D TVS

Pearson Correlation	Difference between 2D and 3D measurements	Sig. (one- tailed)
IO width	0.345	0.001

## Discussion

In our daily practice, 2D TVS is essential in defining the relation between the lower placental edge and the IO in cases of low-lying placenta and placenta previa. This relation is fundamental in differentiating these types and for decision-making regarding the mode of delivery in such risky cases [14, 15]. In a previous case report, different measurements for EOD were reported by two sonographers [16]. However, the reproducibility of EOD measurement by 2D TVS and the inter-observer variability were not studied. Moreover, the conflicting results of the different studies about the cutoff EOD above which vaginal delivery can be attempted in these cases raises the suspicion of the inaccuracy of 2D TVS measurement of the EOD [10, 12, 13].

**Fig. 9:**
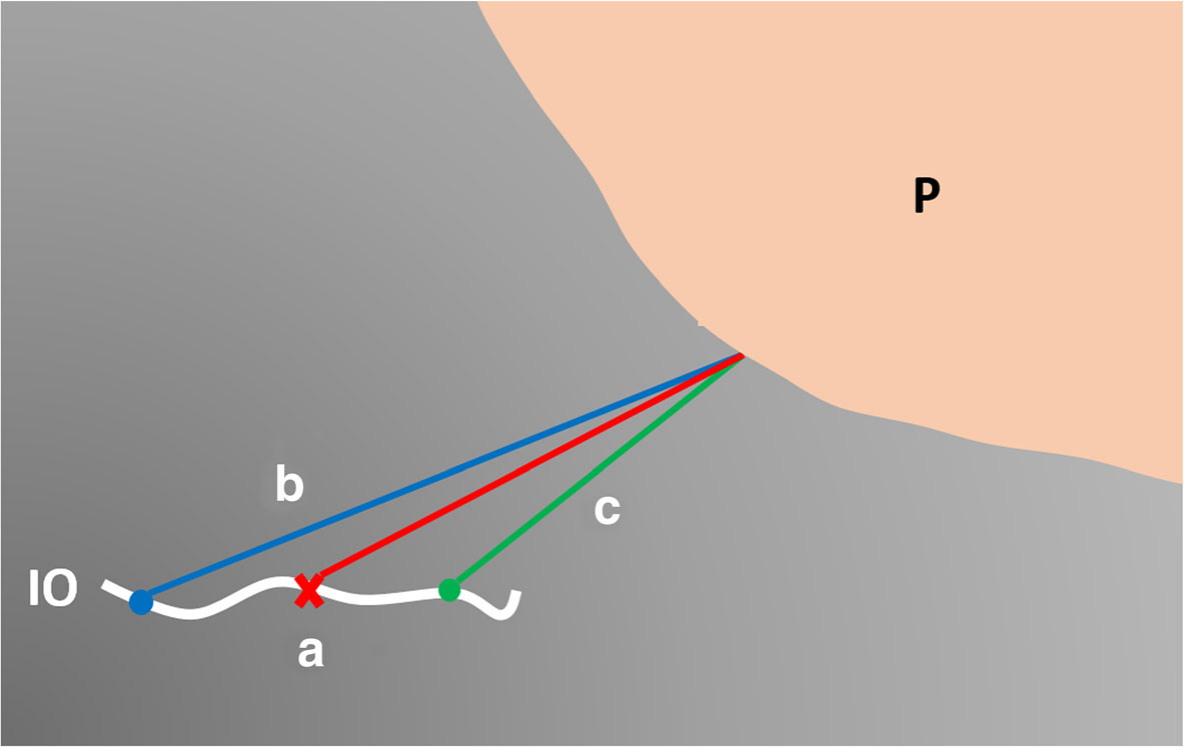
Accurate measurement of EOD from the center of IO to the nearest point of lower placental edge (red line, a). When shifted farther from the placental edge, the EOD is longer (blue line, b). When shifted nearer to placental edge, the EOD is shorter (green line, c). P: Placenta; IO: Internal os

Theoretically, using 2D TVS, we localize the IO as the uppermost point of the cervical canal in the midsagittal view of the cervix. This would be the case if the cervical canal was tubular in shape and the IO has a pinhole appearance. However, in all cases, we found that the cervical canal and the internal os appeared as a slit, in the axial view of the cervix, surrounded by an oval hyperechogenic area representing the cervical mucosa (previously described by *Simon and his colleagues* as an “oval patch”) [16]. So, it is impossible to guarantee that the view in 2D examination of the cervix is strictly midsagittal, which may lead to errors in measurement of EOD; being nearer or farther from the placental edge (Fig. 9). Moreover, upon rotation of the vaginal probe to get the shortest EOD, both the IO and the lower placental edge must be visualized all through the movement, which becomes impossible upon reaching 90° lateral rotation on both sides. This is specifically important in cases of laterally located placentas. Therefore, another method was needed for more accurate spatial localization of the midpoint of the IO, and for simultaneous visualization of the IO and the lower placental edge during the rotation all around the IO to get the shortest EOD accurately.

The new method of EOD measurement by 3D TVS in the current study has achieved these goals. We could accurately localize the midpoint of the IO, and by positioning the reference point at this location, we could rotate the volume all around the IO while visualizing the lower placental edge to measure the actual shortest EOD whatever the placental location was. From a technical point of view, the most important steps were to place the reference point midway in the slit shaped internal os in plane C, and in the lowest level of the lower uterine segment in planes A and B. In plane B, the whole cervical canal was not visualized in all cases after manipulations, as this canal was curved in most cases and not always perpendicular to the lower uterine segment at the level of IO. However, this was not an essential prerequisite to complete the steps of measurement.

In the current study, the mean EOD measured by 3D TVS was significantly shorter than that measured using the 2D TVS, with dramatic increase in number of cases with EOD ≤ 10 mm and cases with EOD 11–20 mm measured by 3D TVS (Table 4). The most likely explanations of this difference are, firstly, the incorrect localization of the midpoint of the internal os (being *farther* from the placental edge) in 2D TVS and, secondly, the inability to simultaneously visualize the IO and the nearest point of the lower placental edge in a laterally located placenta.

As IO width ranged from 6 to 23 mm in this study, this can make a significant difference in measurement when there is marked shift from IO center. This was confirmed by the significant positive correlation between the IO width and the degree of difference between the EOD measured by both methods (Table 7). In seven cases, EOD measured by 3D TVS was longer than that measured by 2D TVS, mostly due to shift from the IO center *towards* the lower placental edge during 2D EOD measurement.

The difference in EOD measurement by 2D and 3D TVS was also related to the placental location. It was highly significant in anterolateral/posterolateral and lateral locations, being the highest with lateral group (Tables 5 and 6). This supports our hypothesis that the ability of 2D TVS to accurately measure the EOD decreases as the placenta is more lateral in location being almost impossible in directly lateral locations.

In their case report, *Simon* et al [16]*,* described a different method of measuring EOD using 3D TVS, and the difference between 2D and 3D measurements was sufficient to shift from planned vaginal delivery to scheduled cesarean section for their case. They used multiplanar, omniView and surface rendered modes to accurately localize the center of the IO and to simultaneously visualize the whole lower placental edge and the IO.

## Conclusions

Two dimensional TVS may not be accurate in measuring EOD in many cases of low-lying placentas, and 3D TVS may increase the accuracy of measurements in these cases. We believe that this new method is simple, precise and easily applied.

Further research is needed to assess the reproducibility of this method in comparison to the standard 2D method, and whether this innovative 3D technique will make difference in decision-making about mode of delivery in cases of low-lying placentas.

## Additional file

Additional file 1: Demographic and ultrasonographic data for the study group. The file includes the relevant demographic data for study cases including age group, BMI, gestational age, parity and previous cesarean sections. Also, ultrasonographic parameters related to the study including placental locations, edge to OS diameters by both 2 D and 3 D US and internal os width. (XLS 39 kb)

## Abbreviations

2D: Two dimensional
3D: Three dimensional
EOD: Edge to os distance
IO: Internal os
TVS: Transvaginal sonography

## References

[1] Cresswell JA, Ronsmans C, Calvert C, Filippi V (2013). Prevalence of placenta praevia by world region: a systematic review and meta-analysis. Trop Med Int Heal.

[2] Dashe JS (2013). Toward consistent terminology of placental location. Semin Perinatol.

[3] Faiz AS, Ananth CV (2003). Etiology and risk factors for placenta previa: an overview and meta-analysis of observational studies. J Matern Neonatal Med.

[4] Hasegawa J, Nakamura M, Hamada S, Matsuoka R, Ichizuka K, Sekizawa A (2012). Prediction of hemorrhage in placenta previa. Taiwan J Obstet Gynecol.

[5] Gibbins KJ, Einerson BD, Varner MW, Silver RM (2017). Placenta previa and maternal hemorrhagic morbidity. J Matern Neonatal Med..

[6] Young BC, Nadel a, Kaimal a (2014). Does previa location matter? Surgical morbidity associated with location of a placenta previa. J Perinatol.

[7] Bhide A, Prefumo F, Moore J, Hollis B, Thilaganathan B (2003). Placental edge to internal os distance in the late third trimester and mode of delivery in placenta praevia. BJOG An Int J Obstet Gynaecol.

[8] Oppenheimer LW, Farine D (2009). A new classification of placenta previa: measuring progress in obstetrics. Am J Obstet Gynecol.

[9] Royal College of Obstetricians and Gynaecologists (RCOG). Placenta praevia, placenta praevia accreta and vasa praevia: diagnosis and management - Green-top Guideline No.27. 2018. https://www.rcog.org.uk/en/guidelines-research-services/guidelines/gtg27a.

[10] Vergani P, Ornaghi S, Pozzi I, Beretta P, Russo FM, Follesa I (2009). Placenta previa: distance to internal os and mode of delivery. Am J Obstet Gynecol.

[11] Al Wadi K, Schneider C, Burym C, Reid G, Hunt J, Menticoglou S (2014). Evaluating the safety of labour in women with a placental edge 11 to 20 mm from the internal cervical Os. J Obstet Gynaecol Canada.

[12] D’Antonio F, Bhide A (2014). Ultrasound in placental disorders. Best Pract Res Clin Obstet Gynaecol.

[13] Ghourab S (2001). Third-trimester transvaginal ultrasonography in placenta previa: does the shape of the lower placental edge predict clinical outcome?. Ultrasound Obstet Gynecol.

[14] Leerentveld RA, Gilberts EC, Arnold MJ, Wladimiroff JW (1990). Accuracy and safety of transvaginal sonographic placental localization. Obstet Gynecol.

[15] Oppenheimer L, Armson A, Farine D, Keenan-Lindsay L, Morin V, Pressey T (2007). Diagnosis and Management of Placenta Previa. J Obstet Gynaecol Canada.

[16] Simon EG, Fouche CJ, Perrotin F (2013). Three-dimensional transvaginal sonography in third-trimester evaluation of placenta previa. Ultrasound Obstet Gynecol.

